# Adaptation to family-based treatment for Medicaid-insured youth with anorexia nervosa in publicly-funded settings: Protocol for a mixed methods implementation scale-out pilot study

**DOI:** 10.1186/s40337-021-00454-0

**Published:** 2021-08-13

**Authors:** Erin C. Accurso, Karen J. Mu, John Landsverk, Joseph Guydish

**Affiliations:** 1 Department of Psychiatry and Behavioral Sciences, Weill Institute for Neurosciences, University of California, San Francisco, CA USA; 2 San Francisco Department of Public, Health Behavioral Health Services, San Francisco, CA USA; 3 Oregon Social Learning Center, Eugene, OR USA; 4 Philip R. Lee Institute for Health Policy Studies, University of California, San Francisco, CA USA

**Keywords:** Adaptation, Family-based treatment (FBT), Implementation, Mixed methods, Hybrid effectiveness-implementation Type 3 design, “Scale-out”, Children and adolescents, Anorexia nervosa, Atypical anorexia nervosa, Publicly-funded settings, Protocol

## Abstract

**Background:**

Family-based treatment (FBT) for anorexia nervosa is an evidence-based treatment, but its effectiveness is untested among socioeconomically disadvantaged and racially diverse youth. Adapting FBT may facilitate “scale-out” for Medicaid-insured youth served in publicly-funded settings and potentially improve outcomes for more diverse populations.

**Methods:**

This mixed methods effectiveness-implementation Hybrid Type 3 pilot study protocol included a planning period in collaboration with the San Francisco Department of Public Health, culminating in a two-day in-person FBT training for 25 therapists in the county, followed by the opportunity to engage in one year of weekly supervision. The training incorporated FBT adaptations intended to improve fit for low-income families within community-based settings. Treatment appropriateness and acceptability will be measured immediately post-training. Following the training, cases referred for FBT will only be assigned to the trained clinicians who voluntarily opted into long-term group supervision. Clinicians treating at least one FBT case during the supervision period will report on implementation, adaptations, and patient weight gain. Finally, semi-structured interviews with clinician participants will be conducted, focused on implementation challenges and facilitators, local treatment adaptations, and overall satisfaction with FBT.

**Discussion:**

Learning about clinician adaptations will advance knowledge about treatment of eating disorders in publicly-funded community clinics, which serve a racially/ethnically and socioeconomically diverse group of youth. This project is designed to accelerate FBT implementation in publicly-funded mental health systems, and inform service improvements for underserved youth with eating disorders.

## Background

Despite a number of evidence-based psychosocial treatments for mental health disorders, implementation is lagging, with a delay of 17 years for treatments to move from research to community-based settings [1] and little regulatory oversight to ensure that evidence-based standards for psychosocial care are met [2]. Implementation science can improve the uptake of effective treatments into routine clinical settings [3]. Economically disadvantaged youth have poor access to evidence-based treatments, and their implementation is more complex because these treatments are often not tested with low-income, racially/ethnically diverse patients served in the public sector [4]. Generally, implementation efforts in mental health treatments have focused on more common concerns (e.g., mood/anxiety, disruptive behavior), which are likely to have the broadest impact. However, implementation science approaches have been applied infrequently to youth with lower base rate disorders, including those with eating disorders.

Eating disorders have the highest mortality rate of any psychiatric disorder due to high suicide rates [5] and medical instability, including cardiovascular, pulmonary, and gastrointestinal complications, as well as electrolyte abnormalities requiring medical intervention [5–7]. Anorexia nervosa (AN) is particularly precarious. Approximately 20% of those with AN require medical hospitalization, and 40% require re-hospitalization within one year [8]. Early, effective treatment is important for AN because of the high mortality rate [9], potential for a chronic course [10], and association between shorter duration of illness and better outcomes [11]. Data indicate that adolescents with atypical AN (AAN), who present at a normal or above normal weight (versus underweight), are just as medically compromised [12]. However, youth with eating disorders do not typically receive evidence-based treatment [13], leaving them more vulnerable to hospitalization.

Family-based treatment (FBT) for adolescents with AN is the most studied treatment for adolescent eating disorders to-date, supported by four randomized trials [14–17]. It is arguably the only adolescent eating disorder treatment meeting the Level 1 “Well-Established Treatment” criteria for dissemination [18]. Implementation of FBT has been confined to specialized eating disorder programs, including settings in national health care systems such as Australia [19] and Canada [20–22], and privately-funded settings in the U.S. [23, 24]. However, there are no published reports focused on “scaling-out” FBT in publicly-funded settings outside of national health care systems, or in non-specialist community-based settings. In the U.S., FBT has been employed primarily with highly educated, White, English-speaking families. Its effectiveness is untested among socioeconomically disadvantaged youth in publicly-funded settings, who are particularly vulnerable in a system that is under-resourced and impacted by health disparities.

While the prevalence of eating disorders in publicly-insured youth is unknown, prevalence rates are similar across racial/ethnic groups [25], and rates of eating disorder behaviors are similar across socioeconomic levels, but increasing at a faster rate in lower socioeconomic status groups [26]. However, there is a media bias insinuating that eating disorders primarily affect affluent White cisgender females. Gender and sexual minorities are also underrepresented in eating disorders research, despite having heightened rates of eating pathology [27]. As a result, clinicians in publicly-funded settings may be unaware that eating disorders are relevant for the populations with whom they work, which leads to lack of training and investment in diagnosing and treating eating disorders [28]. This may be exacerbated for racial/ethnic minority youth, who have lower rates of mental health service utilization for eating disorders than Whites [29].

Fidelity of an intervention (i.e., the extent to which an intervention is implemented as intended) predicts patient outcomes [30–33], but adaptations—rather than strict adherence—may be required to facilitate implementation and sustainability [34, 35]. FBT is inherently flexible, as it relies on caregivers’ expertise about their family to guide recovery. However, some of the core tenets of FBT are difficult to implement in lower-income populations, such as the expectation that caregivers monitor all meals/snacks.

Meta-analyses show that cultural adaptation improves treatment relevance, acceptability, effectiveness, and sustainability [36, 37]. If not adapted, interventions may meet with therapist resistance and have poor organizational fit [38, 39]. Clinicians report intention to “drift” from manualized FBT immediately post-training [40], and actual drift has been observed in real-world practice [13, 41]. While FBT principles have been incorporated into higher level of care programs [42–45], little work has been done to adapt FBT, which is an outpatient treatment, to community-based outpatient settings. Adaptations have been made for at least one new population (i.e., young adults) [46, 47], for cultural reasons (Japan) [48], and to improve fit within a community-based setting [23, 49]. However, adaptations have not been examined for more racially/ethnically and linguistically diverse populations, or for low-income families in publicly-funded settings.

This article describes the implementation study protocol intended to “scale-out” [50] FBT for use by a new population of therapists (community-based clinicians) with a new population of patients (racially/ethnically diverse, lower-income families) in a new setting (publicly-funded clinics). Cultural adaptations are critical for improving service delivery [51–53] because clinicians are more likely to adopt and implement FBT with high fidelity if they perceive the intervention as acceptable for their setting and population [23]. This project will use mixed methods to identify planned and naturally- occurring adaptations to FBT as implemented in publicly-funded clinics, with the long-term goal to increase health equity and improve publicly-funded services for youth with eating disorders.

## Methods/design

The implementation process in this study was informed by Aarons et al.’s [54] dynamic adaptation process (DAP) model, which guides the implementation process in a four-phased iterative model Through active engagement with multiple stakeholders, adaptations are made proactively in a way that honors the core components and/or principles of the evidence-based intervention. Elements of the exploration, preparation, and implementation phases, as guided by the Dynamic Adaptation Process, are described below. The study protocol was designed to measure outcomes during the *implementation phase* (post-training) outlined in the DAP, including implementation outcomes and to a lesser extent patient outcomes. The study design most closely follows Curran et al.’s [55] conceptualization of a hybrid effectiveness-implementation design, which prioritizes the examination of both clinical effectiveness and implementation outcomes, rather than more exclusively focusing on one or the other. Blending design elements of clinical effectiveness trials and implementation trials is viewed as beneficial and efficient, including Type 1 (i.e., examining clinical effectiveness while observing implementation outcomes), Type 2 (i.e., equal value given to examining clinical effectiveness and testing implementation strategies), and Type 3 hybrids (i.e., testing implementation strategies while observing clinical outcomes) [55]. Given this study’s focus on implementation strategies while observing and gathering some more limited data on patient outcomes, this protocol best reflects a Hybrid Type 3 design. Standards for Reporting Implementation Studies (StaRI) were used to guide this report [56, 57].

### Context of the implementation setting

In December 2018, the San Francisco Department of Public Health (DPH) reached out to the first author to work on coordinating a date for FBT training given increasing numbers of youth with AN and AAN presenting for treatment in the county. Prior to the training, DPH administrators identified five publicly-funded mental health agencies that provided intensive services. Each of the agencies was informed about the two-day training and year-long supervision program via email. Three of these programs were targeted for additional outreach due to their strategic geographic location across the county and Spanish language capacity, given county-level data indicating that 60% of eating disorder cases in the year prior to training were Latinx, most of whom were Spanish-speaking. Meetings between two of the authors (ECA, KJM) and directors of the three targeted agencies were held to discuss the logistics of the two-day training and the one-year supervision program.

Directors then discussed the training with clinicians, and one author (KJM) presented at each clinic about the goals of the training and supervision program. A brief clinician survey completed at these meetings indicated minimal exposure to eating disorders and enthusiasm for serving a new population. Clinicians revealed reservations about whether FBT could be applied to a Medicaid-insured population, given that it had been largely tested in university-based medical centers or specialized community-based settings. An invitation to the training was extended by DPH administrators to a sixth agency the day prior to the training in order to balance the number of “internal” agencies and community-based agencies attending.. All study procedures were approved by the IRB at the University of California, San Francisco.

#### Two-day FBT training

In total, 25 clinicians from six agencies in San Francisco County attended the two-day in-person FBT training in September 2019, including clinicians from the three targeted agencies. The first and second authors (ECA and KJM) led the training, in partnership with local expert FBT clinicians providing publicly-funded services, followed by the opportunity for clinicians to engage in one year of weekly FBT supervision. Languages spoken by clinicians who attended the training included Spanish, Cantonese, Mandarin, and Russian. The first half-day provided a background on eating disorders, including assessment across the spectrum of eating disorders. The remainder of the first day and second day were devoted specifically to FBT for AN and AAN consistent with the FBT manual [58], incorporating didactic presentations, role plays, and discussion [58]. It also incorporated several adaptations designed to improve fit for low-income families. Table 1 provides an overview of content adaptations that were planned during the pre-implementation phase [59]. Ultimately, the first author led decisions on modifications based on her expertise in FBT and knowledge of the clinical population, including feedback from clinicians during the training—to the extent that modifications preserved core elements and functions of the intervention. In addition to modifications of training content, a more global change was made to the training itself, in order to improve clinician-reported intervention acceptability and appropriateness and increase clinician satisfaction with training. The first half-day of standard FBT training would typically review FBT efficacy research findings. Instead, our training provided approximately three hours of training in the assessment of eating disorders (given that our clinician population had limited experience in eating disorders), with brief research presentations on eating disorders in publicly-insured populations and the evidence base for FBT. Further, time was reserved at the end of the training to address clinician concerns about implementation and to collaboratively discuss further adaptations. This is in contrast to standard FBT training, where trainers respond to questions about adaptations by reassuring trainees that novice FBT clinicians often have initial concerns about the feasibility and acceptability of particular intervention components but actively discouraging any departures from the manualized intervention.

**Table 1:** An overview of the key tenets of family-based treatment and planned adaptations for Medicaid-insured youth and their families

Key tenets	Description	Nature and description of the planned potential adaptations	Rationale for adaptation
Agnostic view on the potential causes of EDs	No assumptions are made about eating disorder causes. The cause(s) cannot be determined for a particular individual and are not needed to inform steps towards recovery, but rather might detract from the need for urgent action towards swift recovery	*Tailoring/tweaking*: Additional emphasis on assessing and acknowledging other issues outside the eating disorder to minimize likelihood that the family feels dismissed or misunderstood by the clinician, while emphasizing that most of the critical issues unrelated to the eating disorder cannot be effectively addressed while the patient is malnourished	Increase the acceptability and appropriateness of the intervention at the clinician and patient/family level (enabling greater cultural sensitivity for Latino, Asian, and Black/African American patients/families)
Externalizing the illness	The eating disorder is viewed as having “taken over” the patient around issues related to food/eating, shape/weight, and physical activity, with the patient having little (if any) control over eating disorder-related thoughts, feelings, or behaviors. Externalizing the illness is critical to increase compassion and reduce criticism/blame towards the patient	*Tailoring/tweaking*: Given potentially less parental supervision and greater expectations for self-sufficiency/independence and greater need for their child to be able to achieve independence faster, training emphasized finding repeated opportunities to externalize the illness. Given varying levels of mental health literacy, medical analogies were emphasized as potentially more relatable and concrete ways to assist with externalization and build compassion for why their child may not want to recover (or why they would struggle to recover without support, even if motivated)	Increase appropriateness of language at the patient/family level due to higher levels of mental health stigma and barriers mental health service engagement, as well as possibly greater parental expectations for children to take responsibility for their own health
Nonauthoritarian therapeutic stance	Therapist serves as a consultant to the family (eating disorders expert) while family is the expert on their child; therapist guides the family through treatment but does not tell them exactly to how support their child to recover (e.g., not providing meal plan or directives on how to manage return to exercise, once medically cleared)	*Tailoring/tweaking*: Clinicians (given their advanced education and position) may have more difficulty achieving a nonauthoritarian stance with socioeconomically and racially/ethnically diverse families (who face systemic challenges, such as language barriers, racism) than with more privileged families, where the perceived power differential might be smaller. Because families might perceive clinicians as unnecessarily withholding expertise per the manual, this adaptation allowed for offering more concrete guidance and support to caregivers on the treatment process (when needed). The therapeutic stance was maintained by eliciting caregivers’ thoughts about which suggestions might work for their family, if any, and inquiring about other ideas that might work	Increase acceptability and feasibility for clinicians who are used to being more directive; increase acceptability and appropriateness for patients/caregivers who may need and/or want more concrete guidance
Parental empowerment and alignment	Clinicians convey confidence in caregivers' capacity to effectively manage the recovery process (e.g., longstanding knowledge of their child, success in feeding this child or other children previously). Caregivers are not to blame for the disorder and are the most important part of the solution. It is essential that they are aligned so that the eating disorder does not divert their efforts	*Tailoring/tweaking*: For caregivers who had been separated from their child for a period of time, particularly if only recently reunited, this adaptation proactively advised caution in using caregivers’ history of effective feeding practices with or knowledge of their child (when unhelpful) to empower caregivers. Instead, clinicians could focus on caregiver strengths in navigating other challenges. In addition, clinicians were trained to be more available to support caregivers in navigating systems (medical care, school) and the way in which they could do this to support caregiver empowerment, rather than undermine it. In addition, caregiver roles and cultural beliefs around caregiver responsibilities are more explicitly discussed and addressed to assist with parental alignment	Increase appropriateness and acceptability for patients/families, particularly those who may have experienced extended separations and/or for whom caregiver responsibilities are shared with multiple caregivers. Increase cultural sensitivity and acceptability for caregivers who may experience systemic and linguistic barriers in advocating within health and school systems, or caregivers with highly defined responsibilities/role by gender or other personal characteristic
Pragmatic and exclusive focus on eating disorder symptoms	Early exclusive focus on interrupting eating disorder behaviors for prompt weight restoration and return to physical health, to the exclusion of other problems (e.g., depressed mood, trauma, social withdrawal), the treatment of which is delayed until eating disorder treatment and weight restoration is well underway	*Loosening structure*: Additional emphasis on assessing and acknowledging other issues outside the eating disorder, in order to ensure that the family does not feel dismissed or misunderstood by the clinician, while emphasizing that most of the critical issues unrelated to the eating disorder cannot be effectively addressed while the patient is malnourished. Additional latitude was given to address other issues that might interfere with parental success in renourishment (e.g., housing, employment, finances, caregiver health), and thoughtfulness around how to address other issues (social work referral if possible, or scheduling additional sessions to assist with other issues)	Increase the acceptability and appropriateness of the intervention at the clinician and patient/family level, given socioeconomic challenges faced by populations with lower incomes and multiple psychosocial stressors, and clinicians’ training in addressing these issues (and their importance for professional satisfaction)

#### Planned potential adaptations

Parental empowerment is a central tenet of FBT. Caregivers are charged with managing eating disorder behavior in the first phase of treatment, including all decisions around nourishment (e.g., what foods, when, how much) and personal responsibility for supervising all meals and snacks. These activities demand a level of parental involvement that is often not feasible for single caregivers, or caregivers with demanding work schedules, inflexible work hours, or limited financial resources. As an adaptation, clinicians were trained to empower caregivers to be resourceful in making a meal/snack supervision plan while being more flexible in brainstorming who could provide supervision. Although the manual is explicit that siblings should not assist with supervising meals, a sibling’s role in supporting the patient warrants a more nuanced discussion when families are already depending on older siblings in a parenting capacity. Brainstorming might also include participation from extended family members, family friends, or neighbors as part of the system supporting renourishment efforts.

FBT discussions would also include problem-solving around how to manage snacks and lunch during school hours. Ideally, one caregiver would be available to meet their child for lunch at school, or alternatively caregivers would identify a staff member (nurse, counselor, teacher) who could supervise meals/snacks at school. Rather than empowering caregivers to manage these conversations independently, training allowed for clinicians to empower caregivers by more directly facilitating or supporting caregivers in navigating these conversations and included greater emphasis how to collaborate with school personnel to aid in monitoring lunch/snacks (e.g., requesting accommodations for meal/snack supervision within school setting).

In FBT, there is an expectation that caregivers may contribute in different ways to renourishment efforts, but that parental alignment around their approach is critical, and the importance of balanced contributions so that one caregiver does not carry full responsibility. The adapted training discussed cultural differences in parenting and gender roles in the family, with explicit discussion of caregiver roles in the home and around parenting, and thinking about how to work within an existing system or discussing a reorganized family system needed to support their child. The adapted training emphasized the balance between active engagement from all caregivers and realistic expectations about what level of engagement might be possible. In manualized FBT, sessions are typically rescheduled if any caregiver is absent from a session. Doing so with Medicaid-insured patients and their families risks damaging rapport and signals a lack of respect for the efforts and expense taken by those who did arrive for the appointment. Clinicians were instructed to work with the family that show up for the appointment, but to continue efforts to engage all caregivers, even if engagement meant a brief update by phone during or after sessions.

FBT asks families to “externalize the illness,” an abstract concept meant to help families understand that initially patients have almost no control over their eating disorder. In the adapted training clinicians were trained to invest more time on this concept, as the capacity to externalize the illness is critical to caregiver success in renourishment and in their ability to empathize with their child while remaining firm with the eating disorder. Additional time was spent discussing possible analogies (e.g., eating disorder as a tumor) that might help families understand this concept. These adaptations were intended to increase implementation flexibility without changing core elements related to treatment effectiveness [60].

Finally, clinician reservations about the treatment approach and their concerns about its applicability to their treatment population were directly addressed. Trainers acknowledged that FBT has not been studied in Medicaid-insured populations while emphasizing that FBT has been shown to work more quickly and effectively than alternative treatments [14, 16, 61]. The two local FBT clinicians shared clinical case examples and conveyed confidence about FBT as a treatment approach for Medicaid-insured youth with AN and AAN. Trainers also invited participants to leverage their expertise with this population to adapt FBT for publicly-funded settings. One concern discussed was families’ capacity to engage in weekly office-based therapy, given that agencies provided a mix of office- and home-based services and had extensive experience engaging with families “in the field.” The trainers discussed home-based visits as an adaptation, but several clinicians from different agencies raised concerns that home-based visits might undermine the importance of treatment. Rather, they believed that having families come into the office (rather than clinicians going to the family) would assist families in prioritizing treatment, lend structure and containment to visits that would have been difficult outside of the office, and help manage safety concerns (e.g., access to a provider who could take patient vitals, if indicated). Clinicians discussed how they decided only to deliver dialectical behavior therapy [62] in the office, for similar clinical and safety reasons. Clinical supervisors present at the meeting reached consensus to generally abstain from engaging in home-based visits given these concerns.

### Participants and procedures

Immediately post-training, clinicians will report on appropriateness and acceptability of FBT as an approach for Medicaid-insured youth with AN or AAN. These are key when considering cultural adaptations [63]. Clinician recruitment, with a goal of recruiting 10 clinicians, is in progress. Given the pilot nature of this study and county size, target enrollment is 10 clinicians in order to achieve data saturation for qualitative research. Each clinician who opts into group supervision will receive a copy of the FBT manual [58] and be invited to participate in the study. The recruitment target was also influenced by practical considerations, including resources required to prepare a behavioral health county to identify and refer eating disorder cases. Supervision will occur weekly for approximately one year post-training. Naturally occurring ad hoc adaptations (i.e., those not planned in the initial training) will be discussed on an ongoing basis during supervision. For FBT sessions conducted during the one-year supervision period, clinicians will report on implementation of FBT techniques, note adaptations, and measure patient weight.

### Post-training assessment

Immediately following the two-day training, clinicians will complete quantitative measures.

*FBT Knowledge Assessment (FBT-KA)*. The FBT-KA is an 18-item multiple-choice measure co-developed by the first author that assesses knowledge about FBT for AN and AAN and reviewed/approved by one of the treatment developers.

*FBT Attitude Scale (FBT-AS)* [40]. The FBT-AS is a 20-item measure of clinician attitudes towards FBT. The FBT-AS has good reliability and validity, including predicting intent to change practice to be more consistent with FBT [40].

*Treatment Evaluation Inventory Short Form (TEI-SF)* [64]. The TEI-SF is a 9-item clinician-reported measure of treatment acceptability with good reliability and validity. Instructions requested that clinicians rate each item with respect to how they felt about using FBT for eating disorder behaviors in the context of anorexia nervosa or atypical anorexia nervosa. Generic language in the items was modified (e.g., *this treatment*, *problem behavior*, and *child* were replaced with *FBT*, *eating disorder*, and *client*.

### Active treatment and post-supervision assessment

Clinicians in the one-year supervision cohort who have an active FBT case will complete additional measures. Regardless of whether they have an opportunity to treat an FBT case, they will be invited to participate in a qualitative semi-structured interview as detailed below. Other outcomes, including an economic evaluation, were outside the scope of this pilot study.

*De-identified patient data*. Clinicians will provide de-identified patient/caregiver demographics, diagnosis, session dates, and weights/heights taken by the clinician in the context of treatment.

*FBT Therapeutic Techniques Scale (FBT-TTS)*. [23] The FBT-TTS is a clinician self-report measure of FBT technique implementation completed after each FBT session through six months. Alongside their reported use of FBT techniques, clinicians will be asked to record in real-time any adaptations they make to FBT.

*Qualitative semi-structured interviews*. Approximately one year after FBT is initiated with the first referred case, the first author will conduct semi-structured interviews with clinician participants to learn about implementation challenges and facilitators, naturally occurring treatment adaptations, including rationale and perceived impact on outcome, and overall satisfaction with FBT. Open-ended interview questions will be guided by the five-part, multi-level Consolidated Framework for Implementation Research (CFIR) [65] and the Capability, Opportunity, Motivation, Behaviour (COM-B) model [66]. CFIR considers characteristics of the intervention, outer setting, inner setting, characteristics of the individuals involved, and the implementation process, whereas COM-B focuses on how capability (physical and psychological), opportunity (physical and social), and motivation influence behavior change. The interview guide will be piloted and revised prior to conducting the first interview and will continue to be refined as needed throughout data collection.

### Statistical analysis

The post-training measures will be analyzed using SPSS to describe FBT-related knowledge (FBT-KA), attitudes (FBT-AS), and treatment acceptability (TEI-SF). Measures completed during treatment, will also be analyzed and summarized using SPSS, including self-reported implementation of FBT techniques (FBT-TTS) and patient data. Exploratory, descriptive statistics will be used to examine quantitative data on attitudes and acceptability, given the cohort design with a small sample, which is not sufficiently powered to examine change over time.

Semi-structured qualitative interviews will be transcribed and uploaded into qualitative analysis software (Dedoose [67, 68], exploring themes about treatment acceptability, barriers and facilitators to implementation, and resulting adaptations. Stirman et al.’s [59] Framework for Reporting Adaptations and Modifications-Expanded (FRAME) will be used for categorizing changes into contextual modifications (e.g., changes to format or setting) or content modifications (i.e., changes to “how” or “what” intervention content is delivered). These modifications will be evaluated on the extent to which they represent fidelity-consistent and fidelity-inconsistent adaptations [69]. Trained staff will independently review and code interview transcripts to identify and abstract themes following standardized procedures. Each interview will be reviewed by two researchers, from which a list of emerging themes and codes will be developed. These codes will then be independently applied to each interview by both researchers. Coding meetings will be held during the coding process to identify and codify new themes and resolve coding discrepancies. Qualitative findings on implementation and adaptations will be integrated with quantitative data on the implementation, treatment acceptability, and attitudes towards FBT. Preliminary outcome data will be presented.

## Discussion

This hybrid protocol is designed to “scale out” standard FBT (training and implementation)—adapting training and the intervention itself for clinicians in publicly-funded community settings working with more diverse service populations, with the ultimate goal of increasing access to acceptable and appropriate evidence-based eating disorder treatment for underserved youth. Given that eating disorders are increasingly recognized in a range of service populations [26], adapting evidence-based eating disorder treatments for use in community-based clinics is essential. Data collection is ongoing, currently focused on completing semi-structured interviews with clinician participants to learn about implementation challenges, acceptability of FBT, and local treatment adaptations. Studying how evidence-based treatments are adapted to improve their fit in real world settings or for diverse service populations is important to advancing the science of adaptation in implementation [70]. These adaptations will inform a web-based training in “real world” FBT, which is currently under development. Online training has significant advantages over traditional in-person training as an accessible, flexible, scalable, and sustainable method of training [71, 72]. Online training has been successfully disseminated for evidence-based eating disorder treatments previously [73, 74] and its advantages over standard face-to-face training extend to under-resourced systems with high clinician turnover treating low base-rate disorders such as eating disorders.

### Limitations

This is one of the earliest efforts to study implementation of a treatment for Medicaid-insured youth with eating disorders, but there are several limitations. First, this study includes a limited sample of clinicians treating patients within one county due to practical constraints of preparing a system of care to manage eating disorder patients in coordinated interdisciplinary care. Second, due to the changing context of care with the onset of the Covid-19 pandemic, this team decided that it would not be feasible to engage families in an observational research study when it was evident from supervision that families were struggling to engage in mental health treatment due to pandemic-related challenges. As a result, we modified the protocol to collect de-identified patient data, rather than prospectively collecting more comprehensive patient and family outcomes. Third, and also related to the pandemic, we did not collect session recordings due to the challenges of recording clinical sessions in a virtual environment, including the fact that clinicians were using personal, non-encrypted devices to conduct sessions. As a result, we collected self-report data on implementation, including adaptations, without being able to directly observe and code clinician behavior. Finally, this study is limited by its traditional resource-intensive approach to training (i.e., in-person training conducted over multiple days with follow-up supervision), which cannot be reasonably scaled up.

### Impact

This project will develop a pathway to accelerate implementation efforts in under-resourced publicly-funded mental health systems and inform service improvements for high-risk youth with AN and AAN seeking treatment in community-based settings. Learning about clinician adaptations will advance knowledge about how to treat eating disorders in publicly-funded community clinics, which serve youth who are more racially/ethnically diverse youth than those with whom the interventions have been tested to date. Given the potential benefits of telehealth over traditional in-person services for specialty mental health, lessons on how clinicians additionally adapted FBT in order to adjust to providing treatment via telehealth to lower-income families in the context of the pandemic may be particularly informative. Additionally, we will explore stakeholder attitudes towards FBT and facilitators and barriers to implementation in these settings. Building on the learning from this study, we aim to refine the adapted treatment model to better fit Medicaid-insured youth with eating disorders and settings in which they are served. Studying implementation challenges in the “real world” may also help to identify further refinements to FBT that could improve outcomes more broadly for youth eating disorders across sociodemographic levels and settings. Further, this study may provide context for some of the contextual challenges of implementing interventions in publicly-funded settings (e.g., administrative time needed to access resources, including treatment manuals). A byproduct of this implementation effort may be greater investment in improved screening and treatment of eating disorders, particularly if administrative staff and clinicians trust that these patients—once identified—can be successfully treated with a model that fits well with this population and these settings. Ultimately, this line of research aims to improve outcomes, promote health equity, and support future implementation efforts for Medicaid-insured youth with eating disorders.

## Data Availability

Not applicable.

## Abbreviations

AN: Anorexia nervosa
AAN: Atypical anorexia nervosa
FBT: Family-based treatment
DAP: Dynamic adaptation process
StaRI: Standards for reporting implementation studies
DPH: Department of Public Health
CFIR: Consolidated framework for implementation research
COM-B: Capability, opportunity, motivation, behavior
FRAME: Framework for reporting adaptations and modifications-expanded
